# Does Intensive Land Use Contribute to Energy Efficiency?—Evidence Based on a Spatial Durbin Model

**DOI:** 10.3390/ijerph19095130

**Published:** 2022-04-22

**Authors:** Haiqian Ke, Bo Yang, Shangze Dai

**Affiliations:** 1 Fanli Business School, Nanyang Institute of Technology, Nanyang 473000, China; kehaiqian@whu.edu.cn; 2 Institute of Central China Development, Wuhan University, Wuhan 430072, China; ycloud@whu.edu.cn

**Keywords:** intensive land use, spatial durbin model, energy efficiency, carbon emissions

## Abstract

In order to ensure the safety of cultivated land and promote urban productivity, the Chinese government began to promote intensive land use at the legislative level from 2014. At the same time, China faces problems of carbon emissions and energy, so we need to improve energy efficiency. Therefore, this paper aims to verify the spatial effects of intensive land use on energy efficiency of China from 2009 to 2018. We further use an index system to quantify intensive land use and use chain DEA (data envelope analysis) to quantify energy efficiency. This paper finds that: (1) intensive land use can significantly improve energy efficiency. A 1% increase in the level of intensive land use will increase energy efficiency by 1.3%. (2) The intensive use of land in one city will have a negative impact on the energy efficiency of surrounding cities. The reason is that the intensive use of land in a single city may lead to the transfer of energy-consuming industries to surrounding cities. (3) The impact of intensive land use on the energy efficiency of surrounding cities has negative threshold characteristics, and the negative impact will be weakened as the level of integration of the city increases.

## 1. Introduction

The widespread, severe negative impacts of human activities on Earth’s ecosystems over the past few decades have highlighted the importance of continuous and up-to-date monitoring of ecosystems health [1]. In the context of the “green revolution” sweeping the world and the prevalence of international green trade barriers, only the harmonious coexistence of economic development, energy consumption and the ecological environment can support sustainable development and maintain a competitive advantage. As China has reached crossroads of economic transformation and upgrading, it needs to transform its economic development mode and transform its economic growth momentum. Therefore, under the severe situation of the environmental carrying capacity reaching its upper limit, it is necessary to promote the transformation of the industrial economic growth mode to resource-saving and eco-environmental protection and promote the formation of a development model with higher eco-efficiency [2,3].

In addition, in December 2020, the Chinese government announced a series of new measures for China’s National Independent Contributions, including that carbon dioxide emissions per unit of GDP before 2030 will drop by more than 65% compared to 2005, the proportion of primary energy consumption of non-fossil energy stations will reach about 25%, and forest storage will increase by 6 billion cubic meters compared with 2005, etc. China will strive to reach carbon peak by 2030 and achieve carbon neutrality by 2060. As of 2019, 46 countries and regions around the world have achieved carbon peaks, mainly developed countries. As the world’s largest developing country, China has substantial carbon emissions, accounting for about 28% of the world’s total, and its energy structure is dominated by coal. In 2019, coal accounted for 57.7% of total energy consumption. China’s economic development is still in the stage of stock accumulation, and it still needs to rely on capital stock driven by production investment and infrastructure investment, which, in turn, leads to carbon emissions; energy resource endowments are not abundant enough, and it is difficult to build a diversified and green energy structure. Raising awareness of environmental protection, encouraging green consumption, raising the level of green technology innovation and improving energy efficiency are all ways to mitigate the growth of carbon emissions. Among them, energy efficiency refers to obtaining a higher output under the same energy input, so the improvement of energy efficiency can effectively save the total energy use, thereby reducing related carbon emissions [4]. Improving energy efficiency means that comprehensive innovation and exploration are required to promote a new round of industrial energy revolution. Improving energy efficiency also requires substantial improvements in existing energy production and consumption methods. It is then conducive to promoting new industries, creating new growth points, and realizing a low-carbon transformation of economic development in the process.

Human economic activities use land as the primary bearing space, and a large amount of agricultural land is gradually transformed into non-agricultural land, which has gradually become one of the main factors for the rapid rise of carbon dioxide emissions. Additionally, the change of land use is the second most significant factor behind the increase in global atmospheric carbon dioxide content after the burning of fossil fuels [5]. The main reason is that the intensive use of land is through the increase in labor, capital, technology and other inputs to achieve a high-efficiency use of resources and achieve economic development, while also inevitably increasing the total amount of carbon emissions. In addition, China’s increasingly limited land resources have increasingly restricted the direction and speed of urban development and have threatened food security and ecological security. When the cultivation process of the introduced planted food species is complex, the requirements for the living environment are relatively high, so a large amount of land is needed as a test field. At the same time, the development of the renewable green low-carbon energy industry requires large-scale engineering construction as a platform to support it, so it can be widely promoted and applied [6]. Therefore, as a non-renewable resource, land is an important support for economic development and an essential part of the coordinated and sustainable development of China’s economy, society, and ecology. In the process of reducing carbon emissions, China needs strong support from land resources. The Chinese government has also successively issued a series of policies that emphasize the need to promote the formation of a new pattern of high-quality development and protection of land and space. It is proposed to continuously improve the land-saving and intensive-use system, alleviate the contradiction between human and land, reduce the phenomenon of land waste, and form favorable supporting conditions for the realization of sustainable development [7].

## 2. Literature Review and Theoretical Hypotheses

### 2.1. Energy Efficiency

Energy efficiency, or full-factor energy efficiency, has been the focus of research in the field of population, resource and environmental economics in recent years. As early as 1995, the World Energy Commission gave an explanation of the concept of energy efficiency. Energy efficiency can be defined as: “reducing energy input to provide equivalent energy services.” From an economic point of view, Patterson (1996) defines energy efficiency based on the concept of energy consumption per unit of output: energy efficiency refers to the use of less energy to provide the same service or produce the same effective output. Patterson believes that the improvement of energy efficiency should be based on ensuring economic growth, rather than reducing investment [8]. Kilponel (2003) added the concept of environment to the goal of energy efficiency on the basis of predecessors, further enriching the concept of energy efficiency [9]. From early energy efficiency research, the measurement of energy efficiency comes from the ratio of energy consumption to GDP, which belongs to single-factor energy efficiency. As a method to measure factor input and output, single-factor energy efficiency has the characteristics of good data openness, simple calculation, and clear economic significance. However, it ignores the substitution between factors, and in the actual production process, the inputs of production factors can replace each other to a certain extent, and the substitution rate can be expressed by the marginal substitution rate; single-factor energy efficiency does not take into account the externality of energy consumption, and in the process of energy consumption, waste water, waste gas and solid waste will inevitably be generated. Therefore, energy efficiency will be overestimated if single-factor energy efficiency is used [10].

Based on the shortcomings of single-factor energy efficiency, scholars began to look for better indicators to describe the relationship between energy consumption and output. Hu and Wang (2006) made a breakthrough study on the concept of energy efficiency, and they also combined data envelopment analysis to measure total factor productivity [11]. The addition of labor and capital to the input factors changed the analytical framework of previous studies that only considered energy factors, and at the same time introduced the concept of marginal substitution of factors. This method of calculation has also been widely used by later scholars. On the basis that the output remains unchanged, the ratio of the optimal energy input to the actual input can better present the connotation of efficiency because the coordination of different input elements is considered.

### 2.2. Intensive Land Use

The concept of intensive use of land was first proposed by the classical economist David Ricardo in the theory of land rent, and it mainly refers to the increase in capital, labor and other factors input on the land to obtain higher output [12]. However, the input and output of capital and labor in the land is not an eternal, single positive correlation. After the input exceeds a certain range, the output will decrease as the input increases. Therefore, the increase in intensive land use is limited; when the continuous investment of capital and labor in land reaches the point of diminishing economic returns, that is, when the marginal revenue equals the marginal output, the operator will not add additional input. This critical point is the intensive boundary of land use. Land use that reaches the intensive boundary is called intensive land use. Conversely, land use that does not reach the intensive boundary is called extensive use.

Under these constraints, by changing the investment intensity and utilization intensity, the utilization efficiency can be maximized [13]. It emphasizes the sustainable development of intensive land use under the constraints of resource environmental protection. Technological progress and green policy system support are generally considered to be important ways to improve energy efficiency. This is because green emission reduction policies adjust industrial and energy structures to eliminate outdated production capacity and jointly achieve the goal of reducing carbon emissions [14]; technological progress can improve energy efficiency, save energy and increase the development and utilization of clean energy. It is worth noting that the intensive use of land at the urban level can help increase density, cause creative destruction, environmental regulation, and ultimately increase energy efficiency [15].

### 2.3. Intensive Land Use and Energy Efficiency

However, the intensive use of land may have a spatial effect on energy efficiency, that is, the level of intensive use of land in a certain city will have an impact on the energy efficiency of surrounding cities. Existing research on pollution transfer issues from the perspectives of environmental regulation, foreign direct investment, foreign trade, economic growth, etc., verify the existence of a “polluted paradise” or “pollution refuge” [16]. China is a large developing country. For a long time, due to the huge differences in geographical conditions and resource endowments between regions, there has been a long-term large imbalance in the level of regional economic development. For areas with high levels of development, in order to improve the quality of local economic development and realize the upgrading of the industrial structure, those industries with high pollution intensity and obvious negative externalities of production may be transferred out [17]. However, low-level development areas may have undertaken this part of the industrial transfer due to their economic growth as their primary goal. Therefore, for regions with low development levels, integration into large economic zones or urban agglomerations may face greater ecological environmental challenges while gaining industrial transfer and economic division of labor dividends.

Theoretically, the “Pollution Haven Hypothesis” (PHH) from the international division of labor indicates that, in the process of advancing international economic integration, regions with low levels of development may face the threat of pollution transfer from regions with high levels of development [18]. In other words, when there is a clear gap in the level of economic development between regions, the transfer of industries may be selective due to the inconsistent preference for economic growth and environmental pollution. Those late-developing areas with lower environmental requirements may become “pollution refuges” for pollution-intensive industries [19]. This pollution transfer effect caused by the difference in the level of economic development has been supported by many documents between developed and developing regions [20]. Therefore, the implementation of the intensive use of land in a certain city may lead to the transfer of industries from that city to surrounding cities, thereby improving the energy efficiency of the city and reducing the energy efficiency of surrounding cities.

However, this effect can be reduced to a certain extent. Regional integration effectively reduces the administrative barriers to the flow of factors and commodities, promotes the spatial allocation and integration of industrial resources and plays an important role in coordinating regional economic development and tapping the potential for economic growth through government cooperation between localities (regional integration in this article refers to economic integration, that is, the elimination of artificial factors that hinder the effective operation of the economy). Through mutual cooperation and unification, multiple separate economies are integrated into a large economy, so that goods and factors tend to flow freely in this large economy [21]. In recent years, with the implementation of the new urbanization development strategy, government cooperation within urban agglomerations has continued to deepen, and the integrated construction of urban agglomerations as a space carrier has attracted more and more attention from scholars [22]. This article suggests that spatial integration could be used to explore the inhibitory effect of intensive land use on the energy efficiency of surrounding cities for the following reasons: first, the spatial integration will to a large extent produce the coordinated development of governance capabilities within the spatial scope, which will lead to the coordinated governance of pollution across regions. By jointly carrying out the analysis and assessment of differences in current environmental protection standards, and orderly formulating and revising unified environmental protection standards in the fields of air, water, soil, hazardous waste, noise, etc., the spillover effects of pollution can be greatly reduced; secondly, the overall level of green innovation will be improved through integration, including the construction of a green industry system [23], for instance, jointly build a green technology innovation center and a green engineering research center, implement major green technology research and development and demonstration projects, encourage the National Green Development Fund to increase investment in the twin city economic circle; advocate a green lifestyle, co-build a standardized technical support platform for green cities, improve a unified green building standard and certification system, accelerate the promotion of garbage classification and jointly build a regional integrated garbage classification and recycling network system; carry out green development experiments and demonstrations, etc. [24]. In summary, the question is whether there is a relationship between intensive land use and energy efficiency and whether this relationship shows spatial characteristics. At present, there are few studies focusing on these questions, so this paper will explore the answers to these questions in depth, thereby enriching the research about land intensive use and energy efficiency.

## 3. Data and Methods

### 3.1. Data

#### 3.1.1. Data Selection

To figure out the relationship between intensive land use and energy efficiency, the following variables are selected in this paper:

(1) Explained variable (energy efficiency, *Ene*): Referring to Patterson (1996) and Fan et al. (2020) [25], the input variables selected in this article are labor (unit is thousands, measured by the number of employees), capital (unit is RMB million, estimated according to the method of Lu Fei et al. [26] and energy (unit is thousand tons of standard coal); the output variable is GDP (unit is one hundred million yuan). This study uses the chain network DEA to quantify energy efficiency [27,28], so each city above the prefecture level is a decision-making unit *DMU_i_*, assuming that there are s (*s* = 1, 2, ..., *S*) stages in the whole process, the input variables and output variables of each stage are *I_i_^s^* and *O_i_^s^*, respectively, and satisfy *I_i_^s^*∈*R_+_^αs^* and *O_i_^s^*∈*R_+_^βs^*; the intermediate variable of the *s* stage and the *s* + 1 stage is set to *P_i_^(s,s+*1*)^*, and it satisfies *P_i_^(s,s+*1*)^*∈*R_+_^γ(s,s+*1*)^*, where *α*, *β* and *γ* represent the number of input variables, output variables and intermediate variables, respectively, *α* = 1, 2, ..., *x*, *β* = 1, 2, ..., *y*, *γ* = 1, 2, ..., *z*. *λ^S^* is the model weight, *w^S^* is the weight variable of the *s_th_* order in the whole process, and *λ^S^*∈*R_+_^n^*, *μ^s^*^−^ and *μ^s^*^+^ are the slack variables of the input variable and the output variable [29,30], respectively; the goal of the network envelope analysis model can be expressed as *θ*:

(1) $$\theta =min\frac{\sum_{s=1}^{S}\omega^{s}[1-\frac{1}{\alpha }(\sum_{x=1}^{\alpha }\frac{\mu_{x}^{s-}}{I_{x_{0}}^{s}})]}{\sum_{s=1}^{S}\omega^{s}[1+\frac{1}{\beta }(\sum_{y=1}^{\beta }\frac{\mu_{x}^{s+}}{O_{y_{0}}^{s}})]}$$

The constraints are:

$$I_{0}^{s}=\sum_{i=1}^{n}\lambda_{i}^{s}I_{i}^{s}+\mu^{s-}\;O_{0}^{s}=\sum_{i=1}^{n}\lambda_{i}^{s}O_{i}^{s}+\mu^{s+}\;P^{(s,s+1)}\lambda^{s+1}=P^{(s,s+1)}\lambda^{s}\;\sum_{i=1}^{N}\lambda_{i}^{s}=\sum_{s=1}^{S}\omega^{s}=1\;\lambda^{S},\mu^{S-},\mu^{S+},w^{S}\geq \;0$$

The efficiency of stage *s* can be expressed as:

(2) $$\theta_{s}=\frac{1-\frac{1}{\alpha }(\sum_{x=1}^{\alpha }\frac{\mu_{x}^{s-*}}{I_{x_{0}}^{s}})}{1+\frac{1}{\beta }(\sum_{y=1}^{\beta }\frac{\mu_{x}^{s+*}}{O_{y_{0}}^{s}})}$$

(2) Explanatory variable (intensive land utilization, *Liu*): China currently divides regional land into construction land and non-construction land. The former can be understood as the land that serves productive labor and capital factors and where various factors of production can play a role; the latter refers to other land, which does not have the nature of production. Because (positive) intensive land use refers to the investment of more production factors per unit area of land and the agglomeration of urban internal space, this article selects GDP density, population density, electricity consumption density, employment density, local fiscal expenditure density, and the inverse numbers of urban patch density (the inverse number of indicators that measure the degree of urban decentralization). The calculation method of density is the ratio of the total amount of economic indicators to the area of construction land. The ratio of the total amount to the total urban area is not adopted because many cities have large tracts of land without economic activities, and only the area of construction land is engaged in economic activities. The weighting is divided into subjective and objective methods. This article selects the EVM (entropy value method), that is: first adopt the normalization method for the data (*x_i_−x_min_*)/(*x_max_−x_min_*), then suppose there are *m* indexes that have been normalized, and each index has *n* data, then the entropy value of the *j_th_* item is:

(3) $$-\frac{1}{Ln\left(n\right)}*\sum_{j=1}^{m}\frac{x_{ij}}{\sum_{i=1}^{n}x_{ij}}*Ln\left(\frac{x_{ij}}{\sum_{i=1}^{n}x_{ij}}\right)$$

Information redundancy *d_j_* is the sum of the opposite of entropy and 1, and the weight of the indicator *wA_j_* can be written as: dj∑j=1mdj

(4) $$d_{j}=1+\frac{1}{Ln\left(n\right)}*\sum_{j=1}^{m}\frac{x_{ij}}{\sum_{i=1}^{n}x_{ij}}*Ln\left(\frac{x_{ij}}{\sum_{i=1}^{n}x_{ij}}\right)$$

Following the EVM method above, the final weights given to the above indicators in this article are: 0.1322, 0.2462, 0.1896, 0.1308, 0.2404 and 0.0606. Because, under this index system, the intensive land use index has both positive and negative values, in order to facilitate the analysis of the quadratic term in the heterogeneity analysis part, in this paper, the value obtained under the index system is summed with 1 to obtain an index to measure the level of intensive land use.

**Figure 1:**
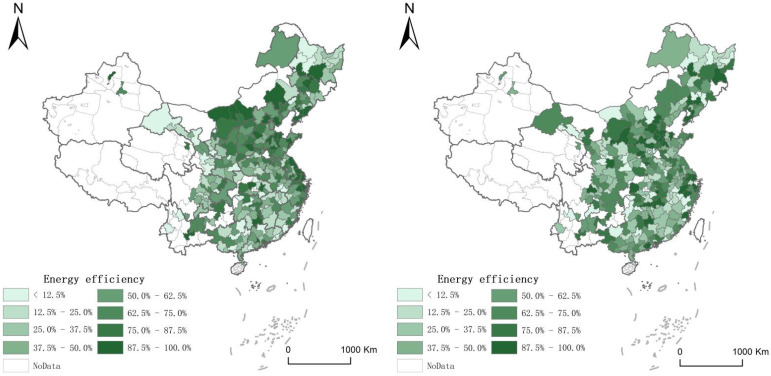
The distribution of the energy efficiency of China in 2009 and 2018.

**Figure 2:**
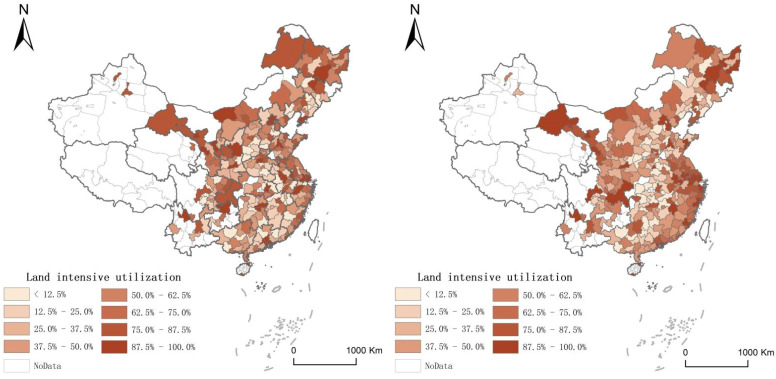
The distribution of land intensive use of China in 2009 and 2018.

We draw maps as Figure 1 and Figure 2 to reflect the distribution of the energy efficiency and the intensive land use of Chinese cities from 2009 to 2018. Due to the data availability, the below maps are based on the majority of Chinese cities.

(3) Threshold variables (spatial integration, *Spa*): the existing literature does not have a method to quantify spatial integration, so this article proposes the level of urban construction integration to measure it. The specific method is to use night light data to connect the geometric center of a certain city with the geometric centers of all neighboring cities. The night light brightness of all county-level administrative units passing through the connection is averaged and normalized for the measurement of spatial integration. If the value is larger, it means that even between the two city centers, the brightness value of the night lights is still very large, which, to a certain extent, indicates that the construction of the city is connected together, and this reduces the cost of transporting goods and commuting people and represents a great deal of spatial integration (two regions can be “surrounding areas” if they border each other).

(4) Control variables: In order to control the impact of other variables on energy intensity, this article selects four control variables: economic development, industrial structure, opening to the outside world and technological innovation. (1) Economic development. Faster economic development leads to more energy consumption, but the development of new technologies and upgrading industries will also lead to improved energy efficiency, thereby saving energy. Therefore, this article uses the logarithm of GDP (*Gdp*) and GDP per capita (*Gpp*) as the control variables [31,32]. (2) Industrial structure. Areas with a higher proportion of the secondary industry may have more carbon emissions. The reason is that industrial production consumes a lot of energy and reduces energy efficiency. Therefore, this article chooses the secondary industry (*Ssr*) and tertiary industry (*Tsr*) as the control variables as the proportion of GDP. (3) Opening up to the outside world. Under normal circumstances, the pace of regional opening up in order to attract foreign investment may cause cities to pay more attention to energy and environmental issues, and the higher the degree of opening up, the more likely it is to introduce clean technologies, which may have a positive effect on energy efficiency [33]. Therefore, this paper chooses the actual utilization of foreign capital as a control variable as the proportion of GDP (*Fdi*) [34,35,36]. (4) Technological innovation: Under normal circumstances, cities with higher technological innovation levels will also show more green technological innovations, which will help save energy and reduce emissions [37], thereby improving energy efficiency factors. This article selects the logarithm of the number of patent applications (*Pat*) and innovation efficiency (*Ine*) of the city as the control variables, and the quantitative method of the latter refers to Ke et al. (2021) [38], and all the variables’ descriptions are listed in Table 1.

#### 3.1.2. Data Source

The quantity of patent applications in this article was manually compiled on the CNKI platform. The rest of the data come from the “China Statistical Yearbook”, “China City Statistical Yearbook”, Statistical Yearbooks of Various Provincial Administrative Units, and “China Energy Statistical Yearbook”. The night light data are from the Visible Light Imaging Linear Scanning Service System (DMSP/OLS) in the U.S. Defense Weather Satellite and Visible Near Infrared Imaging Radiometer (NPP/VIIRS) from the National Polar Orbit Satellite. The sample selected in this paper is 280 cities as the data availability, and the time period is from 2009 to 2018. The missing data are filled in by the difference method (if a city has a missing year, we use the temporal trend of the variable to make it up).

### 3.2. Methods: Regression Model

#### 3.2.1. Benchmark Regression

This paper establishes a panel least squares regression model:

*y_it_ = β_1_x_it_ + βControl_it_ + I + t + ε_it_* (5)

Among them, *y* is the explained variable, which is energy efficiency (*Ene*) in this article, *x* is the explanatory variable (*Liu*), which is the level of intensive land use in this article, and *Control* is the control variable. After Hausman’s test, this article adopts a two-way fixed effects model. Therefore, this article adds dummy variables for year t and individual *i*.

#### 3.2.2. Spatial Regression

This article adopts spatial econometric regression. The spatial measurement model is divided into three types: spatial lag model, spatial error model and spatial Durbin model [38]. The spatial lag model adds the spatial lag term of the explained variable to the general panel data model, indicating that the explanatory variable on a certain spatial unit is affected by the explanatory variable of the adjacent spatial unit; the spatial error model adds spatially related error terms, that is, the error term of a certain spatial unit model is considered to be affected by the adjacent spatial unit model error term; the spatial Durbin model integrates the characteristics of the spatial lag model and the spatial error model. This intensity of the influence of adjacent spatial units is represented by a spatial weight matrix. The spatial lag model, the spatial error model and the spatial Durbin model are as follows:

(6) $$y_{it}=\delta \sum_{j=1}^{N}W_{ij}y_{it}+\varphi +\beta x_{it}+C_{i}+\alpha_{t}+\varepsilon_{it}$$

(7) $$y_{it}=\varphi +\beta x_{it}+C_{i}+\alpha_{t}+u_{it},u_{it}=\rho \sum_{j=1}^{N}W_{ij}u_{it}+\varepsilon_{it}$$

(8) $$y_{it}=\delta \overset{N}{\underset{j=1}{\sum }}W_{ij}y_{jt}+\varphi +\beta x_{it}+\delta \overset{N}{\underset{j=1}{\sum }}W_{ij}y_{ijt}\theta +C_{i}+\alpha_{t}+\varepsilon_{it}$$

where: *δ* is the spatial regression coefficient, which represents the influence of the explained variable *y* of the adjacent spatial unit on the explained variable *y* of this spatial unit (*y* is the explained variable, which is energy efficiency “*Ene*”). If it is significantly positive, it means that the explained variable has obvious spatial overflow. That is, the increase in the variable by one spatial unit within the research scope corresponds to the increase in the variable in other spatial units; *u_it_* is the spatial autoregressive error term; *ρ* is the spatial error coefficient, which represents the influence of the adjacent spatial unit error term *u* on the spatial unit error term *u*; *θ* is the spatial lag term coefficient of the explanatory variable, which indicates the influence of the explanatory variable *x* of the adjacent space unit on the explanatory variable *y* of this space unit; *N* is the number of spatial units, and W is the spatial weight matrix. First, use the LM test to determine whether the spatial lag effect and the spatial error effect are significant, then use Wald or LR test to judge whether the spatial Durbin model can be simplified into a spatial lag model or a spatial error model. Assumption 1: *θ* = 0; assumption 2: *θ* + *λβ* = 0. If assumption 1 passes the significance test, it is considered that the spatial Durbin model can be reduced to a spatial lag model. If assumption 2 passes the significance test, it is considered that the spatial Durbin model can be reduced to a spatial error model [34]. After testing, assumption 1 and assumption 2 are not true, so this article chooses the spatial Durbin model.

#### 3.2.3. Spatial Threshold Regression

Additionally, threshold regression is to test whether the parameters of the sample group divided according to the threshold value are significantly different, and it is often used to study the heterogeneity of the interaction between variables. The threshold regression model developed by Hansen (1999) [39] can endogenously divide the data interval according to the characteristics of the data itself, avoiding the randomness of artificially dividing the sample interval. The relationship between each city level and innovation performance may be nonlinear. Traditional linear regression cannot explain the relationship between the two. The threshold model regression is closer to reality. Therefore, this paper adopts the threshold regression model of Hansen (1999), which is different from the traditional threshold model. The spatial threshold model adopted in this paper adds the spatial lag of explanatory variables and explained variables. First, the following single threshold regression model is set:

(9) $$y_{it}{=\lambda }_{0}{+\lambda }_{1}{w\;\times \;D}_{it}\cdot {I\;(thre}_{it}\leq {\;r}_{1}{)+\lambda }_{2}{w\;\times \;D}_{it}\cdot {I\;(\;thre}_{it}{>\;r}_{1}{)+\lambda }_{3}X_{it}+\gamma \cdot {t+\varepsilon }_{it}$$

Among them, *I* (∙) represents an indicative function, and the value is 1 when the expression in the brackets is true and 0 when it is false. *D_it_* is the core explanatory variable, where *w* × *D_it_* refers to the spatial lag term of the explained variable, *Urbs_it_* is the threshold variable, *X_it_* represents the control variable, and *ε_it_* is the random disturbance term. When *Urbs_it_* ≤ *r*_1_, the core explanatory variable *D_it_* coefficient is *λ*_1_; when *Urbs_it_* > *r*_1_, the core explanatory variable *D_it_* coefficient is *λ*_2_. *t* is the time effect, *λ*_0_ is a constant term and *ε_it_*~(0, σ) is a random interference term. Additionally, Equation (9) only assumes that there is one threshold, but there may be two or more thresholds. Due to space limitations, the double and more threshold tests will not be repeated.

To estimate these variables we mentioned above, we used software named STATA 15. This software is a general-purpose statistical software package developed by StataCorp for data manipulation, visualization, statistics and automated reporting.

## 4. Results

### 4.1. Benchmark Regression Results

Based on Equation (5), Table 2 shows the results of the benchmark regression. The first column is the OLS (ordinary least squares) regression before adding the control variables; the second column is the regression result using the two-way fixed effects model; the third column is the regression result of replacing the T test with D-K (Driscoll–Kraay) standard error, and the purpose is to eliminate the influence of heteroscedasticity and cross-sectional correlation on the regression result; the fourth column is the regression result using the system GMM (Generalized Method of Moment),with two-period lag and three-period lag as instrumental variables. The core of the system GMM model is to use the difference equation and the level equation as an equation system, so that the difference variable and the level variable are mutually instrumental variables for system estimation, so that the parameter estimation is more effective; the fifth column is to remove the extreme value. We remove the samples of the highest 5% and the lowest 5% of carbon emissions and perform regression again, which can eliminate the adverse effects of abnormal values on the results; the sixth column is the use of two-stage least squares regression; we choose the urban topographic undulation as an instrumental variable. The reason is that the higher the terrain undulation, the less the land area that can be used for production and living, and the higher the level of intensive land use. However, the terrain undulation may not directly impact energy efficiency.

In summary, during the study period, it can be found that every unit increase in the level of intensive land use will lead to a corresponding increase in energy efficiency by 1.3 units. Therefore, the intensive use of land is an inevitable requirement for developing a circular economy and a conservation-minded society and an essential guarantee for achieving carbon neutrality and improving energy efficiency. As the concept of sustainable development has become more and more widely accepted by people, the ideology of urban planning has also undergone tremendous changes. Sustainable land resource management has also been promoted to the strategic height of the country by countries worldwide. Especially since the 1990s, this kind of change has been clearly manifested, and some new urban planning ideas and urban land use ideas have appeared. For example, the United States of America is facing the social, economic, and political problems caused by land use. Thus, the USA tries to improve land management, especially reforming planning regulations. It is hoped that the legal function of planning as a weapon to deal with land development issues has become an issue that academia and government are concerned about.

For the control variables, it can be found that: (1) the increase in GDP and GDP per capita can significantly improve energy efficiency, and the reason is that with the growth of the economy, people’s demand for energy saving, emission reduction and a green and low-carbon lifestyle is increasing. In addition, the industrial structure has been shifting to cleaner production with economic growth, which has caused an increase in energy efficiency. (2) The increase in the proportion of the tertiary industry will help improve energy efficiency, and the decline in the proportion of agriculture will also help improve energy efficiency. Therefore, we can find that, based on paying attention to the carbon emissions caused by industry, we should pay attention to the impact of agriculture on carbon emissions and the impact of reduced energy efficiency. (3) The increase in the degree of opening up will not affect energy efficiency. The reason is that although opening up to the outside world can help green technology progress, developed countries may transfer backward, and energy-intensive industries may transfer to China, which is not conducive to energy efficiency. (4) Innovation efficiency significantly enhances energy efficiency, which is similar to the results of Ke et al. (2021) that innovation efficiency can reduce the ecological footprint, so this article will not repeat it.

Based on Equation (5), Table 3 shows the regression based on the eastern, central, western and northeastern regions. China’s eastern, central and western regions have different levels of economic development and ecological civilization construction, and the development goals of green finance should also be formulated from different angles. The eastern region has a more developed economy, a more mature capital operation, and a more complete policy environment. Green finance projects choose to invest and operate in the eastern region. Under the action of mature systems and mechanisms, they can achieve results in a short period of time. With the help of regional economic power, they can give full play to the role of production factors. These advantages cannot be highlighted in the central and western regions. For the eastern region, the improvement of the level of intensive land use has a greater degree of promotion of energy efficiency than other sectors. The reason is that the eastern region is the top priority of China’s economic development, so it carries a large number of economic activities and industrial development. Therefore, it is necessary to strengthen land use regulation in the eastern region to avoid disorderly expansion of land [40].

The difference in regression results between the sections may be related to the differences in the degree of industrialization in different regions. The contradiction between economic growth, scarcity of resources, and environmental damage will exist for a long time, which will be particularly prominent in the stage of rapid industrialization and urbanization. This result is the predicament of rapid economic growth and a manifestation of the underdevelopment of industrial technology. Of course, this has a lot to do with improper development. Resource and environmental problems are definitely not obstacles that cannot be overcome in economic development. Resource and environmental problems encountered in development cannot be solved by stopping development but can only be solved in development.

### 4.2. Spatial Regression Results

Table 4 shows the regression results of the spatial model. In order to reduce the endogenous problems caused by the omitted variables, this paper adopts the spatial dynamic Durbin model. Based on the research results of LeSage and Pace in 2009 [41,42], the total effect represents the average impact of X on all regions, the direct effect represents the average impact of X on Y in this region, and the indirect effect represents the average impact of X on Y in other regions.

It can be found that although the intensive use of land can positively promote the energy efficiency of the region, it will lead to a decline in the energy efficiency of the surrounding areas. The reason is that the intensive use of land in a particular city may cause industries to shift to the surrounding areas. Therefore, when the intensity of environmental regulations increases, the market can guide different regions to make decisions that suit them. For each region, economic incentive-based environmental regulations have flexibility and incentives, which can more effectively mobilize enterprises’ subjective initiative and enthusiasm. At the same time, it should be noted that different regions can flexibly choose the most suitable type of environmental regulation according to their own specific conditions so as to achieve the purpose of protecting the environment while pursuing the maximization of the company’s own interests. For other control variables, it can be found that the signs of their direct effects and indirect effects are similar. Therefore, the impact on the local area and the surrounding area are similar, and it is similar to the benchmark regression part, so we will not repeat it in this article.

### 4.3. Spatial Threshold Regression Results

We incorporated the spatial lag of intensive land use level into the threshold model. Table 4 shows the threshold effect test. It can be found in Table 5 that whether it is a single threshold model, a double threshold model, or a three-threshold model, they all exist significantly. Therefore, this paper uses the above three threshold models to verify the impact of intensive land use on energy efficiency under different spatial integration levels [43,44].

The regression results of the spatial threshold model are shown in Table 6. It can be found that with the improvement of the integration level, the impact of the intensive use of land in a particular city on the surrounding areas has changed from a negative value to a positive value. Only at the level of integration of the space will the intensive use of land cause a decline in energy efficiency in the surrounding areas. Based on this, the state and local governments should pay attention to the sharing of resources, technology, and information in the region to realize the coordinated development of the regional economy and strengthen joint prevention and control in the region. In turn, it will realize the benign interaction and coordinated development of the region, give full play to the scale effect, synergy effect and agglomeration effect of the region, and effectively promote the coordinated governance in the region. When implementing differentiated environmental policy combinations in different regions of our country, governments at all levels should be guided by economic means and market regulation, pay attention to encouraging governments and enterprises to use economic incentive-type environmental regulation policies, and guide different types of industrial enterprises to achieve clean and green development based on the characteristics of economic incentive environmental policies. At the same time, different regions use economic incentives and stimulus measures to promote the transformation and upgrading of different types of industrial enterprises as soon as possible.

Spatial integration is conducive to improving the marketization of resources and environmental factors and the allocation efficiency of resources and environmental factors. For example, the trading market of pollution emission rights will form incentives for enterprises to reduce emissions. Market integration can reduce the transaction cost of environmental protection technology, facilitate the promotion and application of various energy saving and emission reduction technologies, promote the spillover of production technology between regions, narrow the gap of environmental protection technology between regions, and narrow the gap of regional pollution emissions. The integration of infrastructure and public services will improve the sharing and efficiency of public resources and reduce energy consumption and emissions.

## 5. Discussions

As the land use change has been the second largest factor contributing to the increase in global atmospheric carbon dioxide, more and more scholars are considering the need to emphasize ecological and environmental protection or the coordinated development of the two in the process of intensive land use. In the meanwhile, energy efficiency, which plays a vital role in sustainable development of economy, has been the hot topic of research in the relevant field. On this basis, the relationship between land use and energy efficiency has also been concerned recently.

Existing studies have mostly focused on the economic effects brought about by intensive land use and its impact on the environment, and few have directly explored the relationship between intensive land use and energy efficiency from a spatial perspective [45,46,47,48]. However, this paper takes prefecture-level and city-level data from 2009 to 2018 as the research object and examines the complex impact of intensive land use on energy efficiency from a spatial perspective, which will help China realize the development of a larger-scale urban green economy, and has certain research value and application significance. Additionally, this paper has the following limitations, which can be improved in further study: first, this paper studies the impact of intensive land use on energy efficiency based on urban data in China. However, whether this proposition is also applicable in other countries deserves further robustness test. Secondly, this paper uses the DEA method to quantify energy efficiency, which can be quantified in a variety of ways in the future to improve the credibility of the proposition. Thirdly, although the improvement in energy efficiency mitigated the trend of carbon emission growth to some extent, it did not reduce the trend of total increase in carbon emission scale. To what extent the contribution of land intensive use to energy efficiency can continuously reduce carbon emissions is a direction we can study in the future. Finally, this paper does not attempt to use a general equilibrium framework for analysis, so it can be attempted in future studies.

## 6. Conclusions

In order to examines the relationship between intensive land use and urban energy efficiency, contributing to the sustainable development of economy, based on the panel data from 2009–2018, this paper first uses the least squares regression model to examine whether intensive land use contributes to energy efficiency, and then the spatial Durbin model and the spatial threshold model are used to empirically examine the spatial effects of intensive land use on energy efficiency and the changes that occur with the process of spatial integration. The results of our research show that: (1) the intensive use of land can contribute to the energy efficiency positively, as each percentage point increase in the level of intensive land use will increase energy efficiency by 1.3 percentage points. (2) Although the intensive use of land can improve the local energy efficiency of the region, it will have a negative effect on energy efficiency of the surrounding areas because of the transfer of energy-intensive industries to the surrounding areas. Space integration can solve this problem to a large extent. (3) The negative impact of intensive use of land on the energy efficiency of surrounding cities will be weakened when the level of integration of the city and its surrounding areas raises.

## Figures and Tables

**Table 1:** Variables explanation.

Variables	Name	Explanation	Data Source
dependent variable	energy efficiency (*Ene*)	this study uses the chain network DEA to quantify energy efficiency.	Yearbooks of various provincial administrative units, and “China Energy Statistical Yearbook”
independent variable	intensive land utilization (*Liu*)	we select GDP density, population density, electricity consumption density, employment density, local fiscal expenditure density, and the inverse numbers of urban patch density, using EVM method to give weights.	“China Statistical Yearbook”, “China City Statistical Yearbook”, Chinese Basic GIS data.
threshold variable	spatial integration (*Spa)*	the specific method is to use night light data to connect the geometric center of a certain city with the geometric centers of all neighboring cities. The night light brightness of all county-level administrative units passing through the connection is averaged and normalized for the measurement of spatial integration.	Visible Light Imaging Linear Scanning Service System (DMSP/OLS) in the U.S. Defense Weather Satellite and Visible Near Infrared Imaging Radiometer (NPP/VIIRS) from the National Polar Orbit Satellite
control variables	GDP (*Gdp*)	gross domestic product	“China Statistical Yearbook”, “China City Statistical Yearbook”
	GDP per capita (*Gpp*)	gross domestic product per capita	
	secondary industry ratio (*Ssr*)	the proportion of secondary industry in total GDP.	
	tertiary industry ratio (*Tsr*)	the proportion of tertiary industry in total GDP.	
	opening up (*Fdi*)	the proportion of actual utilization of foreign capital in total GDP.	
	patent applications (*Pat*)	logarithm of the number of patent applications	China national knowledge infrastructure
	innovation efficiency (*Ine*)	quantitative method o refers to Ke et al. (2021)	China national knowledge infrastructure, “China Statistical Yearbook”, “China City Statistical Yearbook”

**Table 2:** Benchmark regression results.

Variables	(1) OLS	(2) FE	(3) D-K	(4) GMM	(5) Drop Extremum	(6) 2SLS
*Liu*	1.209 *** (−3.61)	1.321 *** (4.34)	1.514 *** (5.17)	1.412 *** (3.84)	1.732 *** (4.70)	1.318 *** (4.68)
*GDP*		0.183 *** (6.45)	0.246 *** (6.54)	0.287 *** (9.73)	0.203 *** (8.08)	0.262 *** (9.76)
*Gpp*		0.230 *** (4.09)	0.322 *** (4.26)	0.166 *** (5.52)	0.241 *** (5.38)	0.231 *** (4.90)
*Ssr*		−2.152 *** (−6.54)	−2.733 *** (−6.29)	−2.249 *** (−6.16)	−1.875 *** (−5.27)	−2.522 *** (−6.34)
*Tsr*		3.367 *** (10.62)	3.268 *** (11.53)	2.339 *** (9.42)	3.385 *** (8.33)	2.670 *** (9.68)
*Fdi*		−0.211 (−1.09)	−0.308 (−1.17)	−0.084 (−1.20)	−0.107 (−0.99)	−0.151 *** (−1.19)
*Pat*		−0.130 (−0.49)	−0.087 (−0.50)	−0.084 (−0.49)	−0.116 (−0.33)	−0.074 (−0.44)
*Ine*		0.122 *** (6.16)	0.122 *** (5.86)	0.103 *** (4.93)	0.094 *** (5.09)	0.116 *** (4.49)
*Time × Individual fixed effect*	Control	Control	Control	Control	Control	Control
*Constant*	1.215 *** (11.04)	1.192 *** (3.83)	1.052 *** (3.41)	0.656 *** (3.68)	1.248 *** (−3.13)	1.319 *** (−4.21)
*R²*	0.2638	0.7942	0.7942	0.7181	0.5654	0.8013
*Sample size*	2800	2800	2800	1960	2520	2800

Note: *, **, and *** represent significant at the 10%, 5%, and 1% levels, respectively, and the hypothesis test statistics are in parentheses.

**Table 3:** Benchmark regression results of four regions in China.

Variables	(1) Easter	(2) Middle	(3) West	(4) Northeast
*Liu*	1.81 *** (4.40)	1.78 *** (4.27)	1.75 *** (3.30)	1.42 *** (3.39)
*GDP*	0.22 *** (−6.43)	0.19 *** (6.68)	0.22 *** (−9.62)	0.18 *** (−8.53)
*Gpp*	0.15 *** (−5.12)	0.17 *** (4.97)	0.22 *** (−5.40)	0.18 *** (−5.68)
*Ssr*	−2.01 *** (−4.48)	−1.761 *** (−4.69)	−2.20 *** (−5.77)	−2.00*** (−5.83)
*Tsr*	2.46 *** (11.63)	2.429 *** (8.75)	2.41 *** (9.99)	2.04 *** (8.29)
*Fdi*	−0.09 (−1.10)	−0.09 (−1.19)	−0.10 (−1.22)	−0.11 (−1.21)
*Pat*	−0.08 (−0.48)	−0.09 (−0.45)	−0.08 (−0.50)	−0.10 (−0.44)
*Ine*	−0.12 *** (−5.90)	−0.10 *** (−6.27)	−0.09 *** (−6.71)	−0.09 *** (−6.03)
*Time × Individual fixed effect*	Control	Control	Control	Control
*Constant*	0.807 *** (4.64)	1.122 *** (3.29)	1.121 *** (4.50)	1.076 *** (4.73)
*R* ^2^	0.6438	0.7017	0.8056	0.8321
*Sample size*	900	910	570	420

Note: *, **, and *** represent significant at the 10%, 5%, and 1% levels, respectively, and the hypothesis test statistics are in parentheses.

**Table 4:** Spatial regression results.

	Durbin Model			Dynamic Durbin Model		
Variables	Direct Effect	Indirect Effect	Total Effect	Direct Effect	Indirect Effect	Total Effect
*Liu*	2.125 *** (3.22)	−1.242 *** (−4.73)	0.883 *** (2.94)	2.784 *** (4.96)	−1.723 *** (−4.95)	1.061 *** (3.52)
*GDP*	0.272 *** (−6.63)	0.183 *** (−9.06)	0.455 *** (−3.92)	0.193 *** (−9.46)	0.231 *** (−6.13)	0.416 *** (−9.29)
*Gpp*	0.226 *** (−4.47)	0.211 *** (−4.60)	0.437 *** (−4.96)	0.147 *** (−5.57)	0.221 *** (−3.74)	0.368 *** (−3.32)
*Ssr*	−1.942 ** (−5.76)	−2.182 *** (−4.88)	−4.124 *** (−2.64)	−2.536 *** (−4.69)	−1.632 *** (−5.89)	−4.168 *** (−4.55)
*Tsr*	3.011 *** (7.48)	2.453 *** (11.55)	5.464 * (1.91)	3.088 (1.40)	2.760 (1.21)	5.848 ** (2.61)
*Fdi*	−0.125 (−1.04)	−0.094 (−1.24)	−0.219 ** (−2.27)	−0.082 (−0.81)	−0.133 (−1.06)	−0.215 −1.87
*Pat*	−0.082 (−0.44)	−0.083 (−0.44)	−0.165 (−0.89)	−0.103 (−0.51)	−0.082 (−0.39)	−0.175 −0.90
*Ine*	−0.113 *** (−4.30)	−0.114 *** (−6.57)	−0.227 *** (−5.38)	−0.123 *** (−6.26)	−0.121 *** (−5.34)	−0.244 −11.60
*Rho*	10.76 *** (5.61)			8.78 *** (3.63)		
*R* ^2^	0.7804			0.7676		
*likelihood ratio*	1308.272			1295.620		

Note: *, **, and *** represent significant at the 10%, 5%, and 1% levels, respectively, and the hypothesis test statistics are in parentheses.

**Table 5:** Threshold effect test.

	F-Value	*p*-Value	1% Critical Value	5% Critical Value	10% Critical Value
Single threshold	18.937 ***	0.003	15.303	7.955	5.922
Double threshold	40.923 ***	0.007	34.044	18.871	11.045
Third thresholds	−11.530 *	0.090	11.707	−6.286	−12.327

Note: *, **, and *** represent significant at the 10%, 5%, and 1% levels, respectively, and the hypothesis test statistics are in parentheses.

**Table 6:** Spatial threshold effect results.

		(1) Single Threshold	(2) Double Threshold	(3) Third Thresholds
W * Liu	*Threshold variable < δ1*	−0.654 ** (−2.30)	−1.125 ** (−2.02)	−1.432 * (−1.83)
	*δ1 ≤ Threshold variable < δ2*	1.744 *** (4.40)	−1.201 * (−1.92)	−1.532 (−1.39)
	*δ2 ≤ Threshold variable < δ3*	—	3.601 *** (7.61)	2.565 *** (3.07)
	*δ3 < Threshold variable*	—	—	2.278 ** (2.19)
*GDP*		0.272 *** (−9.63)	0.252 *** (−9.11)	0.233 *** (−6.82)
*Gpp*		0.183 *** (−5.37)	0.147 *** (−5.70)	0.215 *** (−4.10)
*Ssr*		−1.895 *** (−4.46)	−2.348 *** (−5.14)	−2.394 *** (−6.65)
*Tsr*		3.273 *** (8.81)	2.127 *** (11.87)	2.034 *** (7.88)
*Fdi*		−0.712 (−1.09)	−0.096 (−1.04)	−0.115 (−1.20)
*Pat*		−0.170 (−0.37)	−0.047 (−0.34)	−0.130 (−0.54)
*Ine*		−0.11 *** (−5.05)	−0.09 *** (−6.65)	−0.10 *** (−6.43)
*Time fixed*		Control	Control	Control
*Individual fixed*		Control	Control	Control
*Constant*		0.692 *** (3.98)	0.781 *** (6.04)	0.882 *** (5.72)
*R* ^2^		0.7348	0.7725	0.7803
*Sample size*		3800	3800	3800
*δ1*		0.1304	0.1475	0.1477
*δ2*		—	0.2843	0.2628
*δ3*		—	—	0.3891

Note: *, **, and *** represent significant at the 10%, 5%, and 1% levels, respectively, and the hypothesis test statistics are in parentheses.

## Data Availability

Due to the confidentiality and privacy of the data, they will only be provided upon reasonable request.
